# CT-based variables of perirenal fat are risk factors for assessing pathological T-stage in patients with clear cell renal cell carcinoma

**DOI:** 10.1080/07853890.2026.2625563

**Published:** 2026-02-09

**Authors:** Hao Guo, Linlin Meng, Lingcheng Zhu, Zhen Zhang, Yumei Zhang, Zehua Sun, Zhongyi Wang, Yang Chen, Jiakang Xu, Jiaqi Li, Heng Ma, Feng Li, Yongli Chu, Xinru Ba

**Affiliations:** ^a^ Department of Radiology, Yantai Yuhuangding Hospital, Qingdao University School of Medicine, Yantai, China; ^b^ Department of Radiology, The Second Hospital, Cheeloo College of Medicine, Jinan, China; ^c^ Department of Radiology, Zibo Central Hospital, Zibo, China; ^d^ Department of Radiology, Peking University Shenzhen Hospital, Shenzhen, China; ^e^ School of Medical Imaging, Binzhou Medical University, Yantai, China; ^f^ Department of Hepatology, Qilu Hospital of Shandong University, Jinan, China; ^g^ Department of Scientific Research, Yantai Yuhuangding Hospital, Qingdao University School of Medicine, Yantai, China; ^h^ Department of Radiology, Yantaishan Hospital, Yantai, China

**Keywords:** Perirenal fat, clear cell renal cell carcinoma, T-Stage, computed tomography

## Abstract

**Background:**

Advances in the treatment of clear cell renal cell carcinoma (ccRCC) toward risk-based therapy intensity modulation have necessitated patient-specific assessments of preoperative T-stage.

**Methods:**

A derivation cohort of 218 ccRCC patients with known pathological results and preoperative biomarker data was used to develop and validate a predictive model for preoperative T-stage. Perirenal fat characteristics were determined by the measurement or evaluation of preoperative CT images. Multivariate logistic regression was used to identify the predictive factors and to develop a predictive model, which was then internally validated using cross-validation and bootstrapping. Model discrimination was assessed using calibration plots. Finally, nomograms were both plotted to visualize model efficiency.

**Results:**

A total of 218 patients (152 males and 66 females) with pathological tumor T-stage (151 low T-stage and 67 high T-stage) were enrolled in this study. Perinephric fat stranding (PFS), RENAL Nephrometry Score (RNS), and preoperative platelet (PLT) counts were independent predictors of a high ccRCC T-stage. The performance of the predictive model built on these variables notably surpasses that of radiologists (AUC: 0.867 vs 0.680; delong test, *p* < 0.001). Internal validation with a K-fold cross-validation (*K* = 10) and a bootstrap method showed good discrimination. The model also demonstrated proper calibration. According to the decision curve analysis, the model was found to be clinically useful (risk threshold probability: 6%–85%).

**Conclusions:**

PFS, RNS, and preoperative PLT provided excellent predictions of a high pathological T-stage for ccRCC patients. This predictive model can serve as a reference to aid clinicians and surgeons in clinical decision-making.

## 1. Introduction

It is widely accepted that obesity is linked to an increased risk for many types of cancer [1]. Clear cell renal cell carcinoma (ccRCC) is one of the most common urological malignancies with annually increasing global incidence, and is also the histological type most closely related to obesity [2]. Empirical evidence suggests that obesity likely affects the clinical course and outcome of ccRCC [3]. Tumor prognosis is strongly correlated with the tumor–node–metastasis (TNM) stage. However, reliable preoperative clinical predictors for accurately predicting the tumor stage of ccRCC are currently lacking. While some studies have demonstrated the potential of abdominal visceral fat as a risk factor for the nuclear grade of ccRCC, the association between abdominal visceral fat and tumor stage remains inconsistent [4,5]. Evidence from an observational study of 83 non-metastatic colon cancer cases indicated that retroperitoneal fat thickness was not associated with a higher stage [6]. A study of patients with early-stage breast cancer (T1 and T2) found a positive correlation between fat surrounding the tumor and the involvement of the axillary lymph node [7]. The inconsistencies among studies could also be related to the complex mechanisms by which obesity increases cancer risk in addition to these methodological differences. Nevertheless, almost certainly the tumour microenvironment was considered to be critical for influencing cancer initiation and progression [8]. A recent study found that tumour cells interacted with perinephric adipose tissue (PAT) to promote ccRCC growth, invasion, and metastasis [9]. Based on this, we propose that more attention should be paid to the perirenal fat than the whole abdominal visceral fat when conducting kidney cancer-related research.

Preoperative blood biochemical data are readily available in oncology stage, and their predictive value has been well established [10–12]. An experimental animal study in mice demonstrated that increased platelet aggregation was related to the tumour stage in colorectal cancer [10]. This might be related to platelet–tumour cell interactions [13]. Likewise, preoperative plasma fibrinogen levels were significantly correlated with TNM stages in gastric cancer [11]. A recent study found that lymphocyte subsets were associated with cancer staging [12]. The coagulation, nutritional and systemic inflammatory status reflected by these blood biochemical indices and cancer were highly linked processes [14,15]. However, current tumour prediction models do not assess for the pathological T-stage in ccRCC patients.

The primary aim of this work was to identify the convenient and reliable predictive factors of pathological ccRCC T-stage combining perirenal fat parameters as assessed *via* computed tomography (CT) with preoperative blood biomarker data. The secondary aim was the development of an innovative a prediction model that can be utilized to identify patients at risk of having higher T-stage tumours.

## 2. Materials and methods

### 2.1. Study design and participants

**Figure 1.**
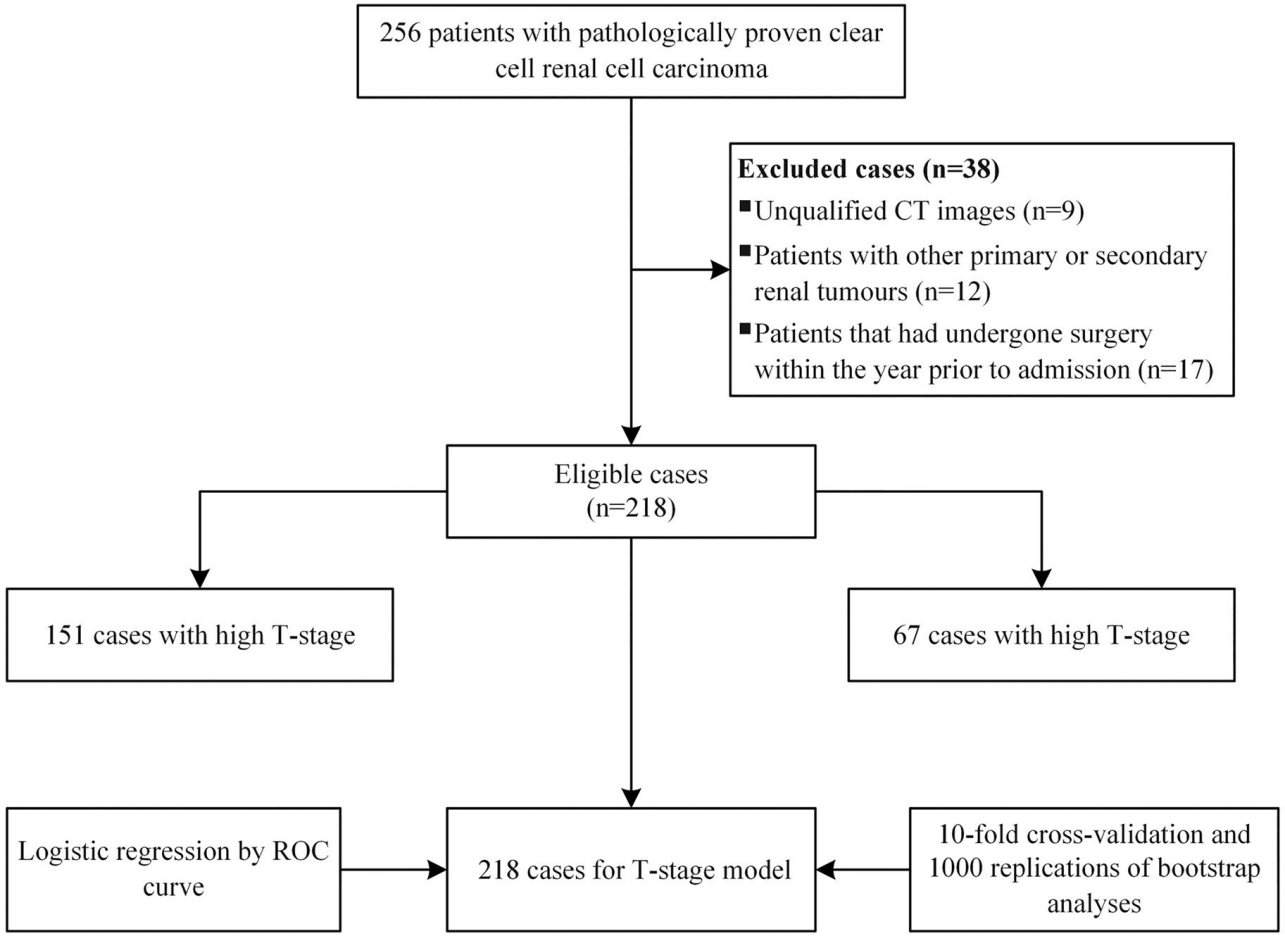
Flowchart of model patients’ enrollment.

Based on the 2013 revision to the Declaration of Helsinki, this retrospective study was conducted. The Yantai Yuhuangding Hospital Ethics Committee approved this study (QDU-HEC-2023141). A waiver of informed consent was granted by the Yantai Yuhuangding Hospital Ethics Committee for this study due to its retrospective nature and the exclusion of patients’ personal information from the analysis. The Picture Archiving and Communication System (PACS) database at Yantai Yuhuangding Hospital contributed the data analyzed in this study, which covered the period from January 2014 to November 2021. CcRCC patients with pathologically confirmed T-stages and no artifacts on preoperative CT images were eligible for enrollment. The following patients were excluded: (a) those with other primary or secondary renal tumours; (b) those who had undergone surgery within the year prior to admission. Finally, 218 patients were enrolled in the study to establish the model. Patient enrollment and the study overview are presented in Figure 1.

### 2.2. Clinical data collection

The data obtained comprised sex, age, body mass index (BMI), preoperative blood routine and biochemical parameters. It was only the results of the last laboratory test performed before surgery that were gathered if multiple tests were performed. The preoperative comorbidities included type 1 and type 2 diabetes mellitus (T1DM and T2DM), thyroid dysfunction, and renal dysfunction. Variables with more than 20% missing were excluded. The tumour-related data including the tumour histology, tumour grading, tumour staging, and RENAL Nephrometry score (RNS) were also collected [16]. According to whether they had high T-stage (T3 and T4) or low T-stage (T1 and T2) ccRCC, patients were divided into two groups [17].

### 2.3. Perirenal fat measurement and evaluation

**Figure 2.**
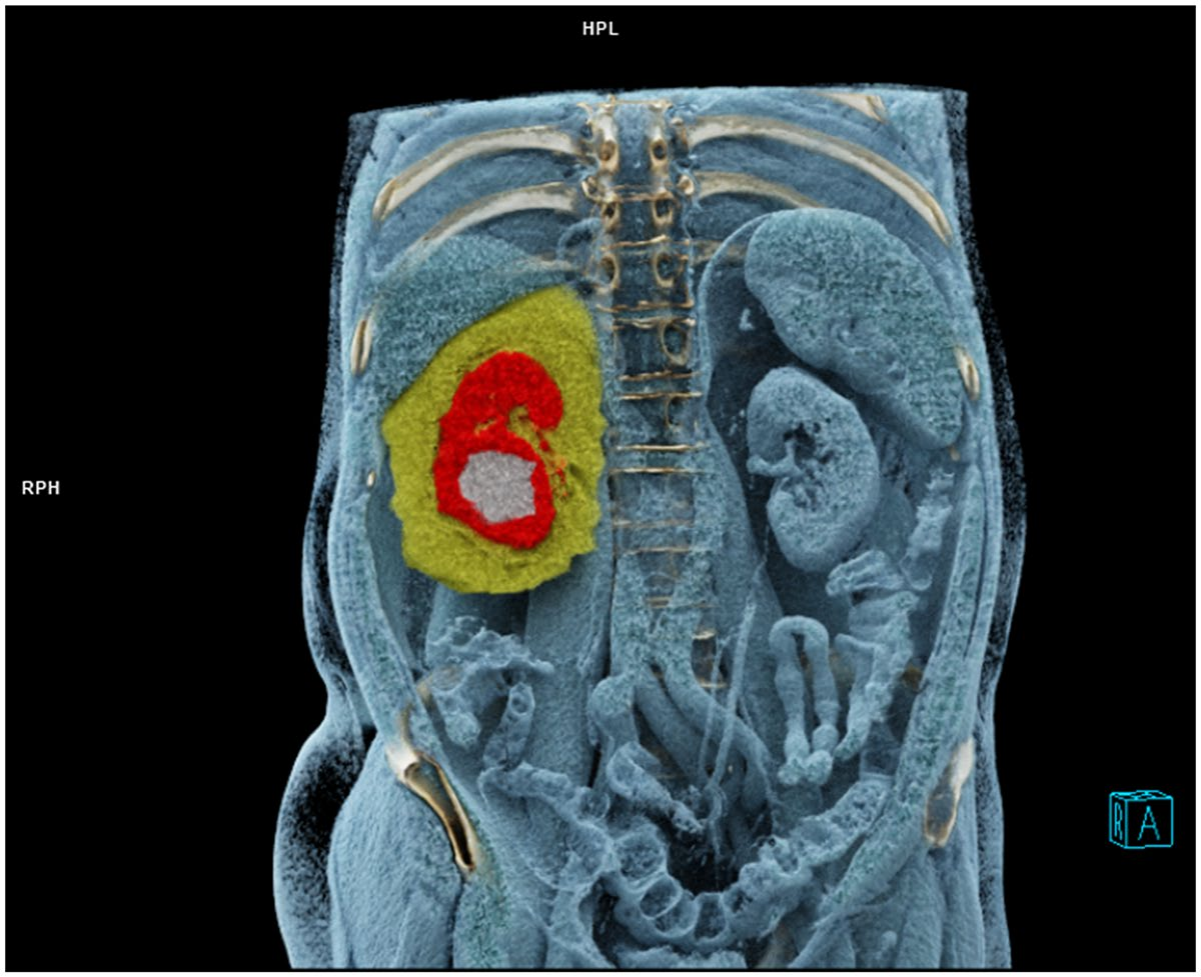
Measurements of perirenal fat were based on the region-growing technique on the syngo.*via* workstation (yellow: perirenal fat, red: normal renal tissue, and white: tumour). The contour of the perirenal fascia was manually drawn on the images of each layer based on the boundaries of the volume of interest (VOI): the cranial and caudal limits of the VOI were set at the most cranial and most caudal image with visible perirenal fascia, respectively, and the volume of perirenal fat (yellow) meeting the Hounsfield unit threshold (−150 to −50) was calculated automatically.

PACS was utilized to extract CT images, which were then imported into the syngo.*via* workstation (VB 30; Siemens Healthcare Solutions, Erlangen, Germany). To avoid the confounding effects on threshold measurements of perirenal fat due to the contrast agent, preoperative plain scan images were used [18]. All images were analysed on the region-growing module of the workstation. For these measurements, the contour of the perirenal fascia was manually drawn on the images of each layer, and the boundaries of the volume of interest (VOI) were defined as follows: the cranial and caudal limits of the VOI were set at the most cranial and most caudal image with visible perirenal fascia, respectively. Finally, an automatic calculation was performed to determine the volume of perirenal fat that met the Hounsfield unit criterion (−150 to −50) (Figure 2). According to Eisner et al. an assessment of perinephric fat thickness at the renal vein level was performed [19]. A measurement of the thickness of the lateral perinephric fat was carried out from the renal capsule to the sidewall in parallel with the renal vein; from the renal capsule to the posterior abdominal wall, the posterior perinephric fat thickness (PPFT) was measured.

**Figure 3.**
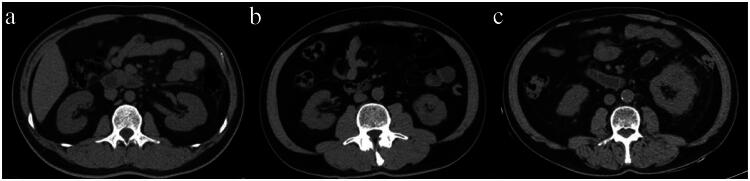
Evaluation of perinephric fat stranding (PFS). (a) None: 0 points. No stranding was found in the perirenal fat area. (b) Mild/moderate: 2 points. Line-like mild stranding was found in the right perirenal fat area. (c) Severe stranding: 3 points. Thick-banded stranding was found in the left perirenal fat area.

On CT images, perinephric fat stranding (PFS) is noted as a linear area of soft tissue attenuation in the perinephric space and is scored according to severity. Perinephric stranding was scored as 0 (no stranding), 2 (thin rim-like mild stranding), or 3 (diffuse, thick-banded severe stranding), as previously described (Figure 3) [20,21]. All the measurements and evaluations were conducted by two independent senior radiologists (radiologist 1, with 21 years of diagnostic radiology experience, and radiologist 2, with 19 years of experience), and uncertainties were resolved by consensus and by contacting other senior radiologists.

### 2.4. Statistical analysis

Percentages and mean (SD) were utilized to describe categorical and continuous data, respectively. The chi-square test and the *t*-test were used to determine the differences in categorical and continuous variables between the low and high T-stage participants. Other continuous variables with median values (interquartile range [IQR]) and those with a non-normal distribution according to the Shapiro-Wilk test were compared using the Wilcoxon test. To identify the independent risk factors for the ccRCC T-stage and develop a prediction model, univariate and multivariate logistic regressions were performed.

Receiver operating characteristic curves (ROCs) were plotted, and AUCs were determined to evaluate the models’ accuracy. Nomograms for predicting the pathological T-stage of ccRCC patients were plotted to visualize and estimate model efficiency. A 10-fold cross-validation was performed to validate the model, with the AUC as the evaluation standard. Bootstrap replications (times = 1,000) were used to assess the model’s stability. Delong tests were performed to assess AUC differences between models. To evaluate the agreement between actual and predicted probabilities, the calibration curve was plotted simultaneously with the Hosmer-Lemeshow goodness-of-fit test. In order to assess the clinical applicability of each model, decision curve analysis (DCA) was performed.

*R* 4.03 software was utilized for all data analyses. A *P* value of less than 0.05 was regarded as statistically significant, and all statistical tests were performed two-sided.

## 3. Results

### 3.1. General characteristics

A total of 218 patients with confirmed ccRCC diagnoses were included in this study (152 males and 66 females; 151 low T-stage patients and 67 high T-stage patients) (Table 1). In regard to biochemical parameters, the preoperative tumour-associated material (TAM) (101.75 ± 15.96 vs. 90.95 ± 13.45, *p* < 0.001), platelet counts (259.00 vs. 226.00, *p* = 0.001), monocyte counts (0.49 vs. 0.43, *p* = 0.006), neutrophil counts (3.98 vs. 3.14, *p* = 0.001), and fibrinogen levels (3.59 vs. 2.90, *p* < 0.001) were significantly higher in the high T-stage cohort than the low T-stage cohort. Conversely, the low T-stage group had higher preoperative high-density lipoprotein cholesterol (HDL-C) than did the high T-stage cohort (1.26 vs. 1.15, *p* = 0.001). Gender and BMI variations across groups were not statistically significant (Table 1).

**Table 1.** The baseline characteristics of all patients in different T-stage cohorts.

Variable	Overall (*n* = 218)	Low T-stage (*n* = 151)	High T-stage (*n* = 67)	*P*
Fuhrman grade, n (%)				
low	158 (72.5)	127 (84.1)	31 (46.3)	**<0.001** ^a^
high	60 (27.5)	24 (15.9)	36 (53.7)	
Site, n (%)				
left	112 (51.4)	69 (45.7)	43 (64.2)	**0.018** ^a^
right	106 (48.6)	82 (54.3)	24 (35.8)	
Sex, n (%)				
female	66 (30.3)	51 (33.8)	15 (22.4)	0.126^a^
male	152 (69.7)	100 (66.2)	52 (77.6)	
Preoperative comorbidities, n (%)				
no	123 (56.4)	85 (56.3)	38 (56.7)	1.000^a^
yes	95 (43.6)	66 (43.7)	29 (43.3)	
PFS, n (%)				
0	77 (35.3)	70 (46.4)	7 (10.4)	**<0.001** ^a^
2	129 (59.2)	80 (53.0)	49 (73.1)	
3	12 (5.5)	1 (0.7)	11 (16.4)	
RNS, n (%)				
low (4 to 6)	26 (11.9)	24 (15.9)	2 (3.0)	**<0.001** ^a^
moderate (7 to 9)	150 (68.8)	113 (74.8)	37 (55.2)	
high (10 to 12)	42 (19.3)	14 (9.3)	28 (41.8)	
Age, year	58.88 ± 10.07	57.51 ± 10.32	61.97 ± 8.79	**0.002^b^**
BMI, kg/m^2^	24.84 ± 4.04	25.16 ± 4.16	23.99 ± 3.59	0.063^b^
PLT, ×10^9^/L	233.00 (196.00, 281.25)	226.00 (192.00, 266.00)	259.00 (216.00, 322.00)	**0.001^c^**
PPFT, cm	1.00 (0.52, 1.58)	1.00 (0.60, 1.50)	1.00 (0.40, 1.60)	0.780^c^
LPFT, cm	1.35 (0.90, 2.10)	1.30 (0.85, 2.10)	1.50 (0.95, 2.15)	0.403^c^
PFV, cm^3^	230.56 (146.36, 353.28)	215.49 (139.03, 325.92)	248.31 (183.48, 415.94)	0.063^c^
WBC, ×10^9^/L	5.84 (4.92, 6.85)	5.73 (4.78, 6.62)	6.36 (5.19, 7.10)	**0.005^c^**
RBC, ×10^12^/L	4.65 ± 0.51	4.70 ± 0.46	4.54 ± 0.61	**0.034^b^**
Neutrophil, ×10^9^/L	3.42 (2.74, 4.42)	3.14 (2.67, 4.22)	3.98 (3.14, 4.66)	**0.001^c^**
Lymphocyte, ×10^9^/L	1.65 (1.33, 2.06)	1.72 (1.36, 2.09)	1.51 (1.26, 2.02)	0.192^c^
Eosinophil, ×10^9^/L	0.09 (0.06, 0.15)	0.09 (0.06, 0.16)	0.10 (0.06, 0.15)	0.963^c^
Basophil, ×10^9^/L	0.03 (0.02, 0.04)	0.03 (0.02, 0.04)	0.03 (0.02, 0.04)	0.814^c^
Monocyte, ×10^9^/L	0.44 (0.35, 0.54)	0.43 (0.34, 0.51)	0.49 (0.36, 0.62)	**0.006^c^**
Fibrinogen, g/L	3.04 (2.69, 3.52)	2.90 (2.60, 3.24)	3.59 (2.99, 4.19)	**<0.001^c^**
TAM, U/mL	94.06 ± 15.00	90.95 ± 13.45	101.75 ± 15.96	**<0.001^b^**
TK1, pmol/L	0.92 (0.41, 1.48)	0.83 (0.36, 1.45)	1.05 (0.53, 1.56)	0.096^c^
HSP90α, ng/mL	66.81 (52.23, 95.33)	64.77 (51.49, 89.58)	78.23 (56.64, 108.25)	0.161^c^
LDH, U/L	185.00 (167.00, 208.00)	184.00 (164.50, 207.50)	188.00 (169.00, 209.00)	0.555^c^
TC, mmol/L	4.77 ± 1.08	4.86 ± 1.05	4.57 ± 1.14	0.069^b^
TG, mmol/L	1.21 (0.81, 1.70)	1.27 (0.84, 1.81)	1.08 (0.80, 1.54)	0.065^c^
HDL-C, mmol/L	1.21 (1.04, 1.43)	1.26 (1.07, 1.48)	1.15 (1.00, 1.30)	**0.001^c^**
LDL-C, mmol/L	2.98 ± 0.86	2.99 ± 0.85	2.95 ± 0.90	0.783^b^
FPG, mmol/L	5.30 (4.75, 5.93)	5.25 (4.74, 5.95)	5.46 (4.79, 5.92)	0.306^c^
Urea, mmol/L	5.34 (4.37, 6.42)	5.33 (4.36, 6.38)	5.51 (4.37, 6.58)	0.541^c^
SCr, μmol/L	63.00 (54.25, 72.00)	62.00 (54.00, 71.00)	65.00 (55.00, 81.50)	0.218^c^
SUA, μmol/L	326.50 (278.25, 405.00)	326.00 (280.50, 397.00)	333.00 (277.50, 427.00)	0.421^c^
Bicarbonate, mmol/L	24.13 ± 2.01	24.25 ± 2.00	23.88 ± 2.04	0.219^b^
eGFR, mL/min	107.97 (91.45, 126.61)	109.21 (94.10, 126.16)	105.71 (80.94, 130.05)	0.226^c^

*p* < 0.05 is indicated by boldface.

^a^ Chi-squared test; ^b^Student’s t-test; ^c^Mann–Whitney U-test.

PFS: perinephric fat stranding; RNS: RENAL Nephrometry score; BMI: body mass index; PLT: platelet; PPFT: posterior perinephric fat thickness; LPFT: lateral perinephric fat thickness; PFV: perinephric fat volume; WBC: white blood cell; RBC: red blood cell; TAM: tumor-associated material; TK1: thymidine kinase 1; Hsp90: heat shock protein 90; LDH: lactate dehydrogenase; TC: total cholesterol; TG: triglycerides; HDL-C: high density lipoprotein cholesterol; LDL-C: low density lipoprotein cholesterol; FPG: fasting plasma glucose; SCr: serum creatinine; SUA: serum uric acid; eGFR: estimated glomerular filtration rate.

### 3.2. Predictors of ccRCC high pathological T-stage

Univariate logistic regression analysis of all preoperative parameters revealed that RNS, PFS, TAM, thymidine kinase 1 (TK1), platelet (PLT) count, triglycerides (TG), HDL-C, fibrinogen levels, monocyte count, neutrophil count, white blood cell (WBC) count, and red blood cell (RBC) count significantly correlated with a high T-stage (Table 2). After adjusting for site, gender, age, BMI, and preoperative comorbidities, preoperative TK1 did not correlate with ccRCC high T-stage. In the multivariate logistic analysis, only PFS (OR 4.057; 95% CI 2.171–7.582; *p* < 0.001), RNS (OR 5.398; 95% CI 2.195–13.275; *p* < 0.001), and preoperative PLT (OR 13.032; 95% CI 2.225–76.310; *p* = 0.004) were independently related to ccRCC high T-stage (Table 2).

**Table 2.** Univariate and multivariate logistic regressions risk factors for ccRCC T-stage.

Variable	Univariate analysis		Multivariate analysis	
	OR (95%CI)	*P*	OR (95%CI)	*P*
Site	0.470 (0.260, 0.850)	**0.013**		
Sex	1.768 (0.908, 3.442)	0.094		
Preoperative comorbidities	0.983 (0.550, 1.756)	0.953		
Age	1.049 (1.016, 1.083)	**0.003**		
BMI	1.000 (1.000, 1.000)	0.143		
Fibrinogen*	69.055 (13.406, 355.696)	**<0.001**		
Monocyte*	4.740 (1.810, 12.414)	**0.002**		
Basophil*	0.913 (0.642, 1.298)	0.613		
Eosinophil*	0.894 (0.526, 1.517)	0.677		
WBC*	4.237 (1.358, 13.223)	**0.013**		
RBC	0.534 (0.298, 0.959)	**0.036**		
Neutrophil*	3.982 (1.710, 9.270)	**0.001**		
Lymphocyte*	0.663 (0.288, 1.526)	0.334		
PLT*	4.370 (1.502, 12.714)	**0.007**	13.032 (2.225, 76.310)	**0.004**
PFV*	1.487 (0.935, 2.364)	0.094		
PPFT*	0.862 (0.652, 1.140)	0.297		
LPFT*	0.959 (0.710, 1.296)	0.787		
PFS	3.003 (1.973, 4.571)	**<0.001**	4.057 (2.171, 7.582)	**<0.001**
RNS	5.581 (2.928, 10.637)	**<0.001**	5.398 (2.195, 13.275)	**<0.001**
TAM	1.054 (1.028, 1.080)	**<0.001**		
TK1*	1.344 (1.007, 1.795)	**0.045**		
HSP90*	1.632 (0.814, 3.272)	0.167		
LDH*	1.565 (0.368, 6.657)	0.544		
TC	0.777 (0.591, 1.021)	0.070		
TG*	0.548 (0.306, 0.982)	**0.043**		
HDL-C*	0.117 (0.031, 0.441)	**0.002**		
LDL-C	0.954 (0.683, 1.333)	0.782		
FPG*	1.890 (0.549, 6.507)	0.313		
Urea*	1.229 (0.432, 3.497)	0.699		
SCr*	1.521 (0.539, 4.287)	0.428		
SUA*	1.535 (0.585, 4.031)	0.384		
Bicarbonate	0.912 (0.787, 1.056)	0.218		
eGFR*	0.601 (0.236, 1.530)	0.286		

*p* < 0.05 is indicated by boldface.

* : log transformation.

BMI: body mass index; WBC: white blood cell; RBC: red blood cell; PLT: platelet; PFV: perinephric fat volume; PPFT: posterior perinephric fat thickness; LPFT: lateral perinephric fat thickness; PFS: perinephric fat stranding; RNS: RENAL Nephrometry score; TAM: tumor-associated material; TK1: thymidine kinase 1; Hsp90: heat shock protein 90; LDH: lactate dehydrogenase; TC: total cholesterol; TG: triglycerides; HDL-C: high density lipoprotein cholesterol; LDL-C: low density lipoprotein cholesterol; FPG: fasting plasma glucose; SCr: serum creatinine; SUA: serum uric acid; eGFR: estimated glomerular filtration rate.

凛冽 呼啸 脸庞

### 3.3. Model construction and validation

**Figure 4.**
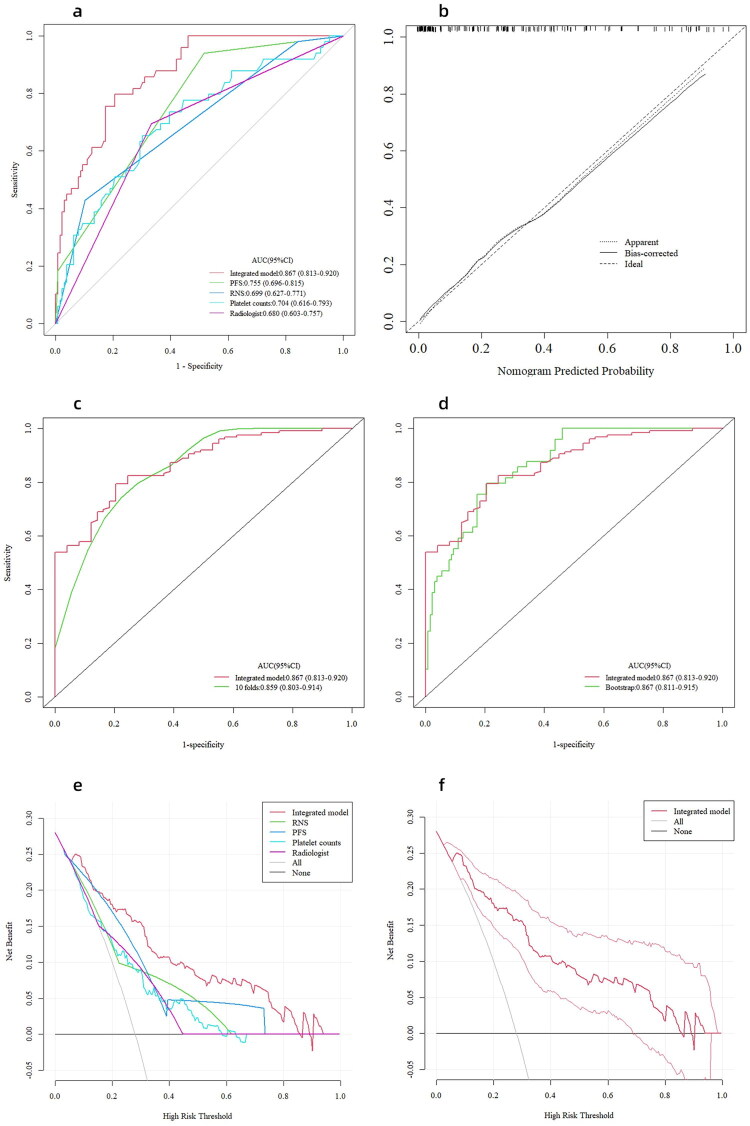
The ROC curves (a), the calibration curve (b), the internal validation of the predictive model (c and d), and the decision curves (e and f). (a) ROC curves for predictive models. The AUC for the combined use of PFS, RNS, and preoperative PLT (red; 0.867 [95% CI: 0.813–0.920]) was significantly higher than the AUC for PFS alone (green; *p* < 0.001), RNS alone (blue; *p* < 0.001), and preoperative PLT alone (light blue; *p* < 0.001). (b) Calibration plot for the integrated model. The shape of the curve on the calibration plots indicated that the model was well-calibrated. (c) 10-fold cross-validation provided similar AUC statistics (AUC: 0.859). (d) Bootstrap sampling (times = 1,000) revealed that the AUC was 0.867. (e) The curve showed that when the probability was between 0.06 and 0.85, the predictive model using all three top-ranking features achieved a higher benefit. (f) The figure depicts the 95% CI of the integrated model.

Based on the c-statistic, the multivariate predictive model, including PFS, RNS, and preoperative PLT, had a discriminative ability of 0.867 (95% CI: 0.813–0.920; sensitivity: 0.796, specificity: 0.794). This value was higher than that of models built with PFS (AUC: 0.755, sensitivity: 0.484, specificity: 0.939; Delong test, *p* < 0.001), RNS (AUC: 0.699, sensitivity: 0.897, specificity: 0.429; Delong test, *p* < 0.001), and preoperative PLT (AUC: 0.704, sensitivity: 0.698, specificity: 0.653; Delong test, *p* < 0.001) alone; meanwhile, the AUC of the integrated model is substantially greater than that achieved by radiologists (0.867 vs 0.680; Delong test, *p* < 0.001) (Figure 4(a)). The calibration plot for the model demonstrated proper calibration (Hosmer–Lemeshow *P* value = 0.800) (Figure 4(b)).

According to a 10-fold cross-validation (AUC: 0.859; 95% CI: 0.803–0.914) and a bootstrap method (AUC: 0.867; 95% CI: 0.811–0.915), the integrated model demonstrated good stability (Figure 4(c,d)).

### 3.4. DCA for the model

The net benefit had a higher risk threshold probability than the baseline, ranging from 0.06 to 0.85, according to the decision curve (Figure 4(e,f)). This also suggested that the model had high clinical applicability. For example, the risk score threshold of 0.28 was at the net benefit 0.158 (0.158 × 100/[0.28/0.72] = 40.63), meaning that the use of the model would reduce over-treatment of 40.63% of non-high-T-stage patients. In our study, this was about 89/218 patients. It is worth noting that the clinical scenario should be considered rather than the best net benefit when selecting the risk probability threshold.

**Figure 5.**
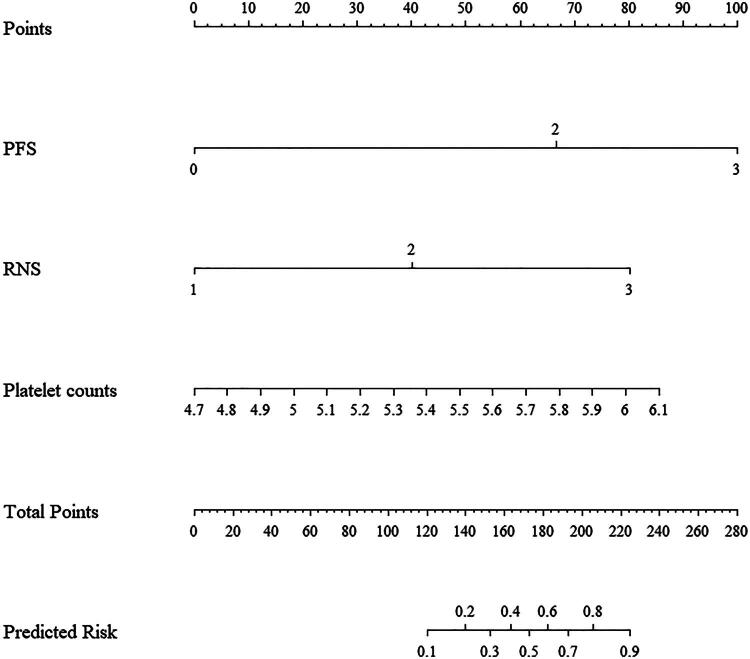
A nomogram for predicting the probability of the ccRCC T-stage. The presence or absence of each parameter indicated a certain number of points. The points for each parameter were summed together to generate a total-points score. The total points correspond to ccRCC T-stage probabilities.

Based on the multivariate logistic regression model, a nomogram was also established for an individual’s estimated risk of having a high ccRCC T-stage (Figure 5). Each patient with a high total score had a greater probability of developing high pathological T-stage ccRCC.

## 4. Discussion

This study demonstrated that PFS, in combination with RNS and preoperative PLT, was better able to predict pathological ccRCC T-stage. Furthermore, to aid clinicians in clinical decision-making, an intuitive and concise predictive model and nomogram was constructed as a reference.

Our results, consistent with previous studies in various types of malignancies, revealed that the preoperative PLT was associated with the pathological T-stage of ccRCC [10]. Even if we restrict the comparison to studies in the kidney cancer-related field, the predictive model has some potential advantages over two previously published models [22,23]. While in a previous study, the authors highlighted how the metabolic tumour volume on ^18^F-FDG PET/CT had adequate discriminative power, but their probative value was obviously limited by their small sample size without accounting for different histological subtypes [22]. Moreover, owing to the high cost and ionizing radiation, PET/CT examinations were not routinely performed in ccRCC patients. Indeed, due to its simplicity and reliability, CT is more widely applicable and can be more readily available in the regular preoperative workup of ccRCC patients. Factors such as PFS and RNS are readily assessable in CT images. Relative to another gene-set-based study, the indicators included in the present study were closer to the real clinical situation [23].

The present study was the first to describe the application of perirenal fat parameters to predict pathological T-stage rather than whole abdominal visceral fat in ccRCC patients. For multivariate analysis, a logistic model was established by utilizing three independent predictors, namely, PFS, RNS, and preoperative PLT, to predict the pathological ccRCC T-stage. Among them, RNS still represented the first assessment of the complexity of renal masses for nephrectomy, reflecting the potential surgical risk [16]. It is now widely used in the preoperative assessment of renal cancer [24]. Furthermore, RNS has been validated to predict the risk of a Fuhrman grade of ccRCC [25]. Chen et al. noted that tumor size and RNS were independent predictors of high-grade in ccRCC [25]. Hence, this indicated its predictive potential in evaluating disease outcomes in ccRCC patients. PFS, as an essential part of Mayo Adhesive Probability (MAP), which concentrates on tumour-environment information, should be taken into account emphatically [21]. MAP calculation comprised two parts: PFS and PPFT [21]. Previous research has also established a correlation between LPFT, perinephric fat volume (PFV), and the outcomes of ccRCC [26,27]. For comprehensiveness, we incorporated LPFT and PFV in addition to PFS and PPFT. In past work, MAP score was found to be a reliable predictor of the presence of adherent perinephric fat (APF), but recently, it has also been revealed to be capable of predicting operative time, intraoperative complications, and postoperative complications in patients who had undergone nephrectomy surgery [21,28,29]. One study found that prolonged operation time and increased estimated blood loss were significantly associated with higher MAP scores during laparoscopic partial nephrectomy [28]. A recent study showed that a high MAP score during a laparoscopic donor nephrectomy may increase the risk of intra- and postoperative complications [29]. Ultimately, PFS was screened as part of the prediction model in the present study. The presence or absence of perirenal fat invasion was strongly associated with the T-stage of ccRCC [30]. PFS might be predictive of the existence of interactions between the tumour and perinephric fat. This could be a preoperative signature of perirenal fat invasion and may explain why PFS was a significant predictor of the T-stage of ccRCC. Notably, PPFT, LPFT, and PFV did not show a similar predictive power. A previous study has shown that browning of the perinephric adipose tissue might be facilitated by parathyroid hormone-related protein released from ccRCC, and that browning adipocytes could either promote or prevent carcinogenesis or advancement through feedback communication [9]. However, the size of adipocytes varies from person to person. Therefore, the morphologic macroscopic changes did not always visible relative to the changes within the perinephric fat [31]. This might explain why the morphological variables of perinephric fat, such as PPFT, LPFT, and PFV, did not demonstrate predictive value in the present study.

Mounting evidence suggests that platelets exert functions in tumor growth, angiogenesis, and metastasis [10,13,32]. Secretory factors released from activated platelets, such as growth-promoting factors, chemokines, and proangiogenic regulatory proteins, can promote tumor cell growth and metastasis [32]. Previous studies have suggested a reliable predictive value of platelets in oncological outcomes [10,15]. However, the utility of platelets for preoperative pathological ccRCC T-stage has not been reported heretofore. The application of preoperative PLT in predicting pathological T-staging in ccRCC patients was reported for the first time in the present study. This facile indicator allows clinicians to be easily and quickly aware of the risks of a higher T-stage in ccRCC patients.

To further verify the accuracy of the model, we performed 10-fold cross-validation in this work. The model demonstrated good performance with cross-validation as well. The calibration curve exhibited reliable linearity and reproducibility. This suggested that our model had a high degree of discrimination and calibration. Furthermore, we also used DCA to evaluate the clinical usefulness of the model. The DCA indicated that our model could provide net benefits across an extensive range of threshold probabilities, relative to models created using each individual variable alone. Comprehensively, the ROC curve, calibration curve, and DCA confirmed that our model was reliable for clinical application. Additionally, we established the first T-stage nomogram for ccRCC patients. Most significantly, the nomogram provided individualized, patient-specific evaluations of the T-stage that can be utilized to classify ccRCC patients according to risk.

We acknowledge several limitations of this study. This model should be recognized as a preliminary tool because, despite the fact that the model’s performance was internally well-demonstrated, the inclusion of an external validation set is often required. Furthermore, we only defined high- and low-T-stages because we preferred to identify those who met risk to a high- or low-degree. On the basis of the results presented here, further subdivision (T-stage classification) could be envisaged. Future prospective multi-centre validation studies with larger sample sizes can address the above limitations.

## 5. Conclusions

In conclusion, PFS, RNS, and preoperative platelet counts may give effective preoperative predictions of pathological T-stage in ccRCC patients. We believe that it will provide a more reasonable tool for ccRCC stage screening compared with invasive methods owing to its high patient practicability. Further investigations will be conducted to externally validate the present integrated model.

## Data Availability

All raw data and code are available from the corresponding author HM (email: mahengyhdyy@163.com) or FL (email: guangzwwf@163.com) on reasonable request.
